# Novel Insights Into Cellular Changes in HPV8-E7 Positive Keratinocytes: A Transcriptomic and Proteomic Analysis

**DOI:** 10.3389/fmicb.2021.672201

**Published:** 2021-09-06

**Authors:** Matthias Kirschberg, Adnan Shahzad Syed, Hanife Güler Dönmez, Sandra Heuser, Astrid Wilbrand-Hennes, Angel Alonso, Martin Hufbauer, Baki Akgül

**Affiliations:** ^1^Medical Faculty and University Hospital Cologne, Institute of Virology, University of Cologne, Cologne, Germany; ^2^Department of Biology, Hacettepe University, Ankara, Turkey; ^3^Cologne Excellence Cluster on Cellular Stress Responses in Aging-Associated Diseases (CECAD) and Center for Molecular Medicine (CMMC), University of Cologne, Cologne, Germany; ^4^German Cancer Research Center (DKFZ), Heidelberg, Germany

**Keywords:** betapapillomavirus, skin cancer, E7 oncoprotein, keratinocyte invasion, fibronectin

## Abstract

Human papillomavirus type 8 (HPV8) is associated with the development of non-melanoma skin cancer. In the past we already delved into the mechanisms involved in keratinocyte invasion, showing that the viral E7 oncoprotein is a key player that drives invasion of basal keratinocytes controlled by the extracellular protein fibronectin. To unravel further downstream effects in E7 expressing keratinocytes we now aimed at characterizing gene and protein/phosphoprotein alterations to narrow down on key cellular targets of HPV8-E7. We now show that gene expression of GADD34 and GDF15 are strongly activated in the presence of E7 in primary human keratinocytes. Further analyses of fibronectin-associated factors led to the identification of the Src kinase family members Fyn and Lyn being aberrantly activated in the presence of HPV8-E7. Phospho-proteomics further revealed that E7 not only targets cell polarity and cytoskeletal organization, but also deregulates the phosphorylation status of nuclear proteins involved in DNA damage repair and replication. Many of these differentially phosphorylated proteins turned out to be targets of Fyn and Lyn. Taken together, by using unbiased experimental approaches we have now arrived at a deeper understanding on how fibronectin may affect the signaling cascades in HPV8 positive keratinocytes, which may be key for skin tumorigenesis and that may also aid in the development of novel therapeutic approaches for betaHPV-mediated cancers.

## Introduction

Cutaneous squamous cell carcinoma (SCC) is the most common metastatic skin cancer, and its incidence is increasing worldwide. DNA damage, as a consequence of excessive UV light exposure, is known to be the main causative factor for the development of cutaneous SCC which arises from a precancerous lesion termed actinic keratosis (Fania et al., 2021). Recent epidemiological data strongly implicate that human papillomavirus (HPV) of genus betapapillomavirus (betaHPV) has a co-factorial role in this process (Hasche et al., 2017, 2018; Hufbauer and Akgül, 2017). The oncogenic potential of betaHPV in skin carcinogenesis was originally identified in patients suffering from the rare inherited disease Epidermodysplasia verruciformis (EV), who have an increased susceptibility to betaHPV infections (Howley and Pfister, 2015). However, betaHPV can also be found in skin cancers of non-EV patients. Particularly, immunosuppressed patients, such as organ-transplant-recipients, also have a higher susceptibility to betaHPV infections of the skin as well as an increased risk of developing SCC compared with healthy individuals (Bouwes Bavinck et al., 2018; Rollison et al., 2019; Smola, 2020).

Despite the more detailed analyses of the viral E6 oncoprotein in keratinocyte transformation, the precise molecular mechanisms by which the E7 oncoprotein may interact with the host cells are still being investigated. We have previously described already that the E7 protein of the oncogenic betaHPV type 8 (HPV8) plays a pivotal role in inducing keratinocyte hyperproliferation and invasion when expressed in primary human adult keratinocytes (PHAK) differentiating in de-epidermalized human dermis (DED)-based organotypic skin cultures (Akgül et al., 2005; Heuser et al., 2016). These keratinocytes display hyperproliferation in the regenerated epithelium and are positive for both cyclin E and p16INK4a, indicating that E7 is able to overcome p16INK4a induced cell cycle arrest (Westphal et al., 2009). In addition, HPV8-E7 positive keratinocytes lose their physiological polarity and invade the underlying dermis (Akgül et al., 2005). Here, downward migration of keratinocytes is facilitated by the degradation of basement membrane components such as collagen VII, collagen IV and laminin V, a process that is paralleled by increased expression of the matrix metalloproteinases (MMP)-1, MMP-8, and membrane-type (MT)1-MMP (Akgül et al., 2005; Smola-Hess et al., 2005). In light of the fact that E7 positive keratinocytes do not exhibit invasive behavior on a collagen type I matrix (Boxman et al., 2001), but do so when cultivated as DED-based organotypic skin cultures, we hypothesized that components of the extracellular matrix (ECM) may be critical in eliciting the invasive behavior of HPV8-E7 positive cells. To address the mechanism by which E7 mediates invasion we tested for a possible cadherin-switch which is commonly seen in invasion-related epithelial-mesenchymal transition. To this end, we examined the E-cadherin and N-cadherin mRNA expression in cells cultured on collagen type IV, laminin V, or fibronectin. Only when HPV8-E7 positive keratinocytes were grown on fibronectin, a reduction in E-cadherin and an increase in N-cadherin levels were detected. These data identified fibronectin as an inducer of the observed cadherin-switch in the presence of HPV8-E7. In subsequent experiments we could detect increased fibronectin levels in peritumoral areas in betaHPV positive SCC as opposed to betaHPV negative tissues. In addition, we showed that E7 positive keratinocytes have elevated levels of integrin α3β1 on their cell surface. Silencing of the α3 chain and the usage of the invasion deficient E7 mutant L23A led to a drastic reduction in the invasive potential of HPV8-E7 cells, thus providing evidence for a direct role of the fibronectin/α3β1 integrin axis in invasion of HPV8-E7 expressing keratinocytes (Heuser et al., 2016). The overall goal of the present follow-up project therefore was to identify further gene expression and protein/phospho-protein alterations using unbiased global transcriptomic and proteomic approaches in order to further pinpoint key downstream cellular targets of HPV8-E7 which may extend our knowledge on the mechanisms underlying betaHPV associated skin carcinogenesis.

## Materials and Methods

### Cell Culture

N/TERT keratinocytes (Dickson et al., 2000) and the spontaneously immortalized human skin keratinocyte cell line PM1 (Proby et al., 2000) were cultivated either in KGM-Gold (0.05 mM Calcium, Lonza, Cologne, Germany) or in RM+ medium (consisting of a 3:1 ratio of Dulbecco’s modified Eagle’s medium (DMEM) and DMEM-F12, supplemented with 10% fetal calf serum (FCS), 1% glutamine, 0.4 μg hydrocortisone, 10^–10^ M cholera toxin, 5 μg/mL transferrin, 2 × 10^–11^ M liothyronine, 5 μg/mL insulin, 10 ng/mL epidermal growth factor, 1 × penicillin–streptomycin mixture) (Hufbauer et al., 2013). The PT67 cell line was maintained in DMEM with 10% FCS and penicillin-streptomycin mixture. All cell lines were cultivated at 37°C and 6% CO_2_.

### Retroviral Expression Vectors and Infection of Keratinocytes

The generation of the pLXSN-based retroviral vectors coding for HPV8 E1, E2, E6, E7, or E6E7 has been previously described (Akgül et al., 2005, 2007; Leverrier et al., 2007). Production of retroviruses and retroviral transduction of N/TERT and PM1 keratinocytes were performed as previously described (Hufbauer et al., 2013). Briefly, keratinocytes were seeded out in 6 cm dishes. Next day, retroviral supernatants were mixed with an equal volume of DMEM in the presence of 5 μg/mL of hexadimethrine bromide (polybrene) and added to the keratinocytes. Spin infection was made by centrifugation for 1 h at 300 × g. Cells were then washed with PBS and cultured further for 2 days. Then, cells were selected against G418 (500 μg/mL) until only infected keratinocytes had survived. In subsequent experiments we used pooled stable cell populations to minimize possible variations due to the randomness of the viral integration site in the cellular chromosomes.

### RTqPCR and PrimePCR

To quantify mRNA levels of cellular genes, quantitative reverse transcription-PCR (RTqPCR) using the LightCycler system (Roche, Mannheim, Germany) was performed as previously described (Hufbauer et al., 2015). The primers used for this study were:

ATF3-fw: TTTGCCATCCAGAACAAGC;

ATF3-rev: CATCTTCTTCAGGGGCTACCT;

GADD34-fw: GCTTCTGGCAGACCGAAC;

GADD34-rev: GTAGCCTGATGGGGTGCTT;

GDF15-fw: CCCGGGACCCTCAGAGTT;

GDF15-rev: CCGCAGCCTGGTTAGCA;

HPRT1-fw: TGACACTGGCAAAACAATGCA;

HPRT1-rev: GGTCCTTTTCACCAGCAAGCT.

The RTqPCR based PrimePCR (Fibronectin-Binding Integrin in Cell Motility H96, BIORAD, Feldkirchen, Germany) was performed in quadruplicate using 96-well plates with pre-spotted primers in each well of the plate. The plates were designed to be analyzed using a Roche LC 480 light cycler, using the SsoAdvanced^TM^ Universal SYBR^®^ Green Supermix. Analyses of these plates were carried out following manufacturer’s instructions.

### DNA Microarrays

The cDNA microarray analyses performed in the Akgül group used total RNA extracted from monolayer cultures of PHAKs in total passage 3 (passage 2 after transduction). The RNA was analyzed in biological triplicates from cells containing either the empty vector control pLXSN or pLXSN-HPV8-E7. Changes in gene expression were measured using the Human Genome U133A arrays according to Affymetrix protocols. Microarray data were analyzed using Transcriptome Analysis Console software (Thermo Fisher Scientific, v4.0.1.36). For analysis of differential gene expression, we applied the following conditions to determine statistically relevant genes: (a) ANOVA test: ebayes, (b) a fold change cut-off of < –2 or > 2, and (c) determination of statistical significance with *p* < 0.05. Gene ontology analysis was carried out using the PANTHER (Protein ANalysis THrough Evolutionary Relationships) database (v14.1). These microarray data files have been uploaded to the Gene Expression Omnibus (GEO) database (GSE133813) (Kirschberg et al., 2019).

The cDNA microarray analyses performed in the Alonso group at the DKFZ were performed with RNA isolated from primary keratinocytes also transduced with pLXSN or pLXSN-HPV8-E7 coding retroviruses. The Affymetrix chip-based experiments and data analyses were performed at the core facility of the German Cancer Center (DKFZ) (Supplementary Table 1). Differentially expressed genes (DEG) from both independent studies were overlapped to minimize bias of the data in respect to genetic variations among keratinocyte donors, cell culture media effects, thus making the results more robust.

### ELISA

The Human GDF15 Quantikine ELISA Kit (RnD systems) was used to measure GDF15 in cell culture supernatants. For this N/TERT-8E7 were cultured on fibronectin and cell culture supernatants were harvested after 4 days. Collected supernatants were treated with protease/phosphatase inhibitors (Protease/Phosphatase Inhibitor Cocktail, Cell Signaling). The ELISA was performed according to the manufacturer’s protocol.

### Western Blotting and Phospho-Kinase Array Analysis

Western blotting was performed as previously described (Marx et al., 2018). Briefly, cell pellets were resuspended in radioimmunoprecipitation assay (RIPA) buffer containing protease inhibitors, incubated on ice for 30 min, sonicated and then centrifuged at 15,000 × g at 4°C for 15 min. Protein concentrations were measured with the Pierce^TM^ BCA Protein Assay Kit (Thermo Fisher Scientific, Dreieich, Germany). SDS gels were transferred upon completion to nitrocellulose membranes, which were then blocked with either 5% bovine serum albumin or 5% dry milk in TBST [10 mM Tris/HCl (pH 8.0), 150 mM NaCl, 0.05% Tween 20]. The primary antibodies used targeted 14-3-3β (sc-59419, clone 60C10, Santa Cruz), ERK1/2 (#4695, clone 137F5, Cell Signaling), Fyn (#4023, Cell Signaling), p-Tyr^530^-Fyn (#PA5-36644, Invitrogen), Lyn (#4576, clone 5G2, Cell Signaling), p-Tyr^397^-Lyn (#orb6340, Biobyrt, Cambridge, United Kingdom), GAPDH-Alexa680 (sc-365062, cloneG9, Santa Cruz). HuT78 whole cell lysates (sc-2208, Santa Cruz) were used as positive controls. Antibodies were incubated for 2 h at RT or overnight at 4°C. Following washing, membranes were incubated with corresponding secondary antibodies (IRDye, LI-COR, Lincoln, Nebraska, United States). Western blots were visualized by employing the LI-COR ODYSSEY FC Imaging system with the Image Studio Ver 5.2. software.

The Human Phospho-Kinase Array Kit (RnD Systems, Minneapolis, United States), spotted with antibodies targeting phospho-sites in kinases associated with malignant processes, was used to identify potentially deregulated kinases in HPV8-E7 expressing keratinocytes grown for 96 h on fibronectin coated tissue culture plates. Cells were rinsed with PBS and then lysed using the provided buffer supplemented with 10 μg/mL Aprotinin, 10 μg/mL Leupeptin, and 10 μg/mL Pepstatin. The lysates were gently incubated at 4°C for 30 min and then centrifuged at 14,000×g for 5 min, before transferring the supernatants into a clean test tube. Samples were then further processed according to the manufacturer’s protocol. Visualization of the membranes and evaluation of individual spot densities were carried out employing the LI-COR ODYSSEY FC Imaging system with the Image Studio Ver 5.2. software.

### Proteomics

Proteomic and phospho-proteomic analyses were performed with total cell extracts from HPV8-E7 expressing N/TERT keratinocytes (N/TERT-8E7) grown for 96 h on fibronectin coated tissue culture plates which were lysed using 8M urea supplemented with protease and phosphatase inhibitors. The whole cell lysates were prepared in triplicates in two separate experiments (*n* = 6) were analyzed at the proteomics core facility of the Cologne Excellence Cluster on Cellular Stress Responses in Aging-Associated Diseases (CECAD) by means of “stable isotope labeling with amino acids in cell culture” (SILAC)-based mass-spectrometry. The protein profile was submitted to the Proteomics Identifications Database (PRIDE) and is available via ProteomeXchange with the identifier PXD026099. Briefly, samples were reduced with DTT (100 mM) and alkylated with 550 mM Iodoacetic acid (IAA) prior to digestion with trypsin and Lys-C overnight at 37°C. Generated peptides were extracted by incubation with increasing amount of acetonitrile, concentrated in a speed-vac and primed prior to LC-MS/MS analysis by the STAGE tip technique (Rappsilber et al., 2003). For liquid chromatography and tandem mass spectrometry (LC-MS/MS), an easy nLC 1,000 (Thermo Fisher Scientific) was coupled to the quadrupole-based Q Exactive Plus (Thermo Fisher Scientific) instrument by a nano-spray ionization source. Peptides were separated on a 50 cm in-house-packed column by a two-solvent buffer system: buffer A (0.1% formic acid) and B (0.1% formic acid in acetonitrile). The content of buffer B was increased from 7 to 23% within 40 min and followed by an increase to 45% in 5 min and a washing and re-equilibration step before continuing with the injection of the next sample. Proteins were considered differentially expressed or phosphorylated under the following criteria: (a) log_2_fold change < –2.5 or > 2.5, (b) *p*-value of *p* < 0.05, and (c) same phosphorylation status in all data sets. Data analysis was performed using the Perseus software suite, KinasePhos2.0 and ShinyGO (v0.61). The differentially phosphorylated protein targets of E7 were imported into the STRING database (Szklarczyk et al., 2019) to identify relevant protein-protein interactions and functional enrichment networks. STRING holds physical as well as functional interaction information derived from high-throughput experimental data, published literature, and computationally predicted co-expression studies.

For secretomic analyses the supernatants of N/TERT-8E7^wt^, N/TERT-8E7^L23A^ as well as empty vector control grown for 96 h on fibronectin coated tissue culture plates (*n* = 6) were harvested and treated with protease/phosphatase inhibitors (Protease/Phosphatase Inhibitor Cocktail, Cell Signaling). Afterward, the supernatants were concentrated using Amicon ultra-15 centrifugation 3K tubes (Merck Millipore, Darmstadt, Germany). Concentrated supernatants as well as a media-only control were then further processed at the CECAD proteomics facility and subjected to mass-spectrometric analysis. The profile of secreted proteins was submitted to the PRIDE Database and is available via ProteomeXchange with the identifier PXD026100.

### Statistical Analysis

Microarray data analyses were analyzed using ANOVA test, setting a log_2_fold change < –2.5 or > 2.5 with a *p*-value of *p* < 0.01. Gene ontology analysis was carried out using PANTHER (v14.1) and ShinyGO (v0.61). RTqPCRs were repeated at least three times in duplicate, and the results are presented as mean ± standard deviation (SD). Statistical significance was determined with an unpaired 2-tailed Student’s *t*-test calculated with GraphPad Prism (Version 9.0). Asterisks shown in the figures indicate significant differences of experimental groups in comparison with the corresponding control conditions (^∗^*p* < 0.05; ^∗∗^*p* < 0.01; ^∗∗∗^*p* < 0.001). Images from immunoblots are from a representative experiment and were qualitatively similar in *n* = 3 experiments. The ANOVA two sample *t-*test was performed to identify statistically significant (*p* < 0.05) peptides in proteomics and the phospho-proteomic data sets.

## Results

### Identification of HPV8-E7 Gene Targets

**FIGURE 1:**
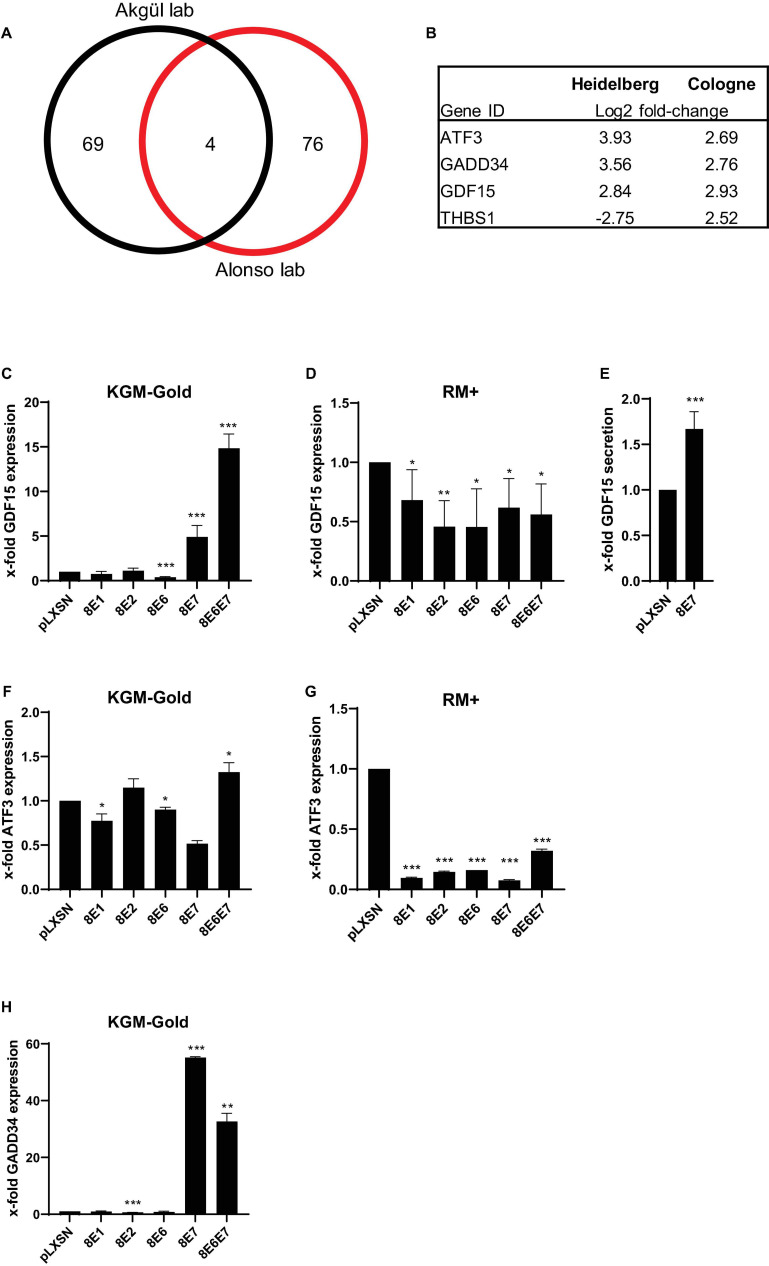
Transcriptional changes in HPV8-E7 positive keratinocytes. **(A)** Venn Diagram comparison of two independent cDNA microarray analyses carried out with PHAK expressing HPV8-E7 in the Akgül lab in Cologne and with PHFK expressing HPV8-E7 in the Alonso lab at the DKFZ in Heidelberg. **(B)** Gene IDs of 4 genes differentially expressed in both array studies. **(C,D)** GDF15 mRNA expression in isogenic PM1 keratinocytes cultured either in KGM-Gold or RM+, respectively. **(E)** GDF15 ELISA performed with cell culture supernatants of HPV8-E7 expressing keratinocytes grown in KGM-Gold medium. **(F,G)** ATF3 mRNA expression in isogenic PM1 keratinocytes cultured either in KGM-Gold or RM+, respectively. **(H)** GADD34 mRNA expression in PM1 keratinocytes cultured in KGM-Gold. RTqPCRs were normalized to HPRT1 mRNA levels (*n* = 3 independent experiments performed in duplicate). Error bars represent standard deviations. Asterisks shown in the figures indicate significant differences of experimental groups in comparison with the corresponding control conditions (*p < 0.05; **p < 0.01; ***p < 0.001).

We previously published a cDNA oligonucleotide microarray (Affymetrix) data set based on HPV8-E7 expressing PHAK which had been grown in KGM-Gold (Kirschberg et al., 2019). In parallel to our own works similar experiments had been carried out by the Alonso group at the DKFZ in Heidelberg using primary human foreskin keratinocytes (PHFK) (Supplementary Table 1). Both experiments were carried out with cells grown on uncoated cell culture dishes. In an effort to identify similarities in gene expression profiles in these HPV8-E7 positive keratinocytes we created an overlap of both sets. After filtering and normalization (log_2_fold-change < –2.5 or > 2.5 and *p* < 0.01) we identified a total of 81 and 73 genes, respectively, in the Cologne and Heidelberg data sets. Interestingly, using these highly stringent cut-off values we still found an overlap of 4 genes (Figure 1A). Three of these genes, namely ATF3 (activating transcription factor 3), GADD34 (growth arrest and DNA damage-inducible protein), and GDF15 (growth differentiation factor–15) were found to be upregulated in both data sets and may therefore be the most relevant target genes of HPV8-E7. In contrast, the THBS1 (Thrombospondin 1) gene showed an opposite regulation which is why this gene was occluded from further analysis (Figure 1B).

In order to further validate our results in cutaneous keratinocytes, the PM1 cell line (Proby et al., 2000) was used which is one of very few keratinocyte lines that can be cultivated in different cell culture growth media. This enabled us to grow keratinocytes under both undifferentiating (KGM-Gold, low-calcium) as well as differentiating (RM+, high-calcium) conditions using the same cell line. Furthermore, in order to underpin the importance of the interaction of E7 with cellular genes the PM1 cells were not only retrovirally transduced with vectors harboring the E7 gene, but also in parallel with constructs expressing E1, E2, or E6.

In the PM1 cell line our experiments showed that E1 and E2 do not have an influence on GDF15 expression. We could, however, confirm a significant effect of HPV8-E7 on GDF15 under non-differentiating conditions, unveiling a fivefold upregulation in gene expression. Most intriguingly, we observed a cumulative effect of E6 and E7 on GDF15 with an up to 15-fold increase in gene expression. Curiously E6 expression alone had appeared to have a slight suppressive effect on GDF15 expression (Figure 1C). Somewhat unexpectedly, these effects on gene expression were greatly diminished, yet still significant under differentiating growth conditions (Figure 1D). Since GDF15 is a member of the transforming growth factor beta superfamily (TGFβ) and therefore a secretory protein we next tested via ELISA whether the elevated GDF15 gene expression may also result in higher protein contents in cell culture supernatants. As shown in Figure 1E, an increase in GDF15 protein levels could also be confirmed in cell culture supernatants of cells grown under non-differentiating conditions. Despite the fact that an upregulation of ATF3 had been predicted in the microarray analyses we could not confirm this by means of RTqPCR. All viral proteins seem to only exert neglible control over ATF3 under undifferentiating conditions (Figure 1F). Under differentiating conditions, however, all virus genes have a strong and highly statistically significant suppressive effect on ATF3 gene expression (Figure 1G). The most profound effect of E7 on gene expression was measured for GADD34 and to a lesser degree for E6E7 positive cells under undifferentiating conditions. The presence of E1, E2, and E6 did not have any effect (Figure 1H). GADD34 gene expression could not be detected at all in isogenic keratinocytes grown in RM+ medium, implying that GADD34 may only play a vital role in undifferentiated basal keratinocytes (data not shown).

### HPV8-E7 Mediated Alterations in Fibronectin Associated Signaling Pathways

**FIGURE 2:**
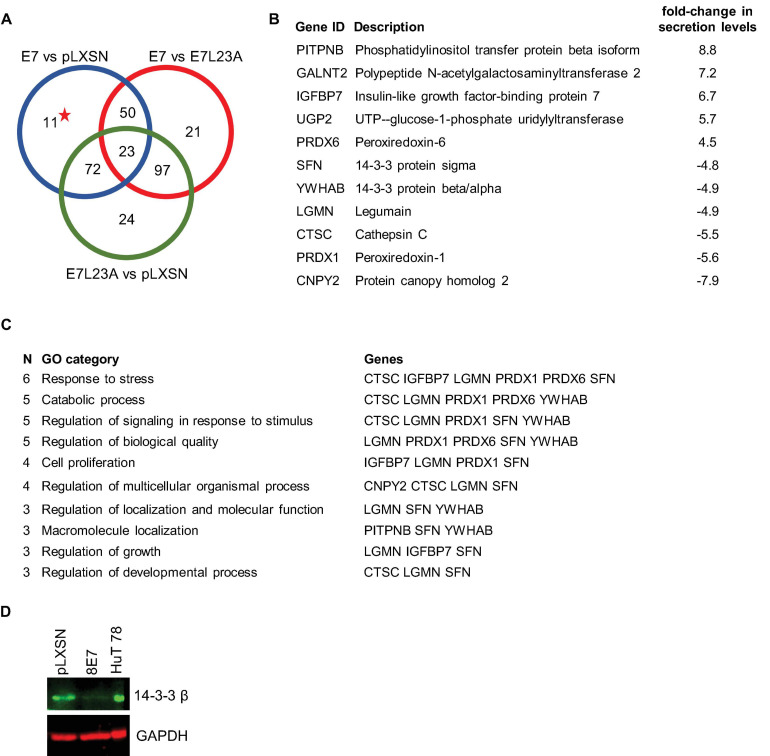
Secretome analysis of N/TERT-8E7^wt^ or N/TERT-8E7^L23A^ grown on fibronectin. **(A)** Venn diagram depicting a three set comparison of differentially secreted proteins taken from N/TERT-pLXSN, N/TERT8-E7^wt^, or N/TERT8-E7^L23A^. The starred number indicates proteins targeted only by HPV8-E7^wt^. **(B)** Description of differentially proteins exclusively secreted by HPV8-E7^wt^ positive keratinocytes as depicted in subfigure A The log2-fold changes of the secretome results from N/TERT-8E7 and N/TERT-pLXSN were compared to calculate the difference. **(C)** Gene ontology analysis of differentially secreted proteins using ShinyGO v0.61. **(D)** Representative immunoblots of RIPA extracts from N/TERTs expressing HPV8-E7 tested for 14-3-3β protein expression (*n* = 3). HuT78 whole cell lysate served as a positive control. Equal loading was confirmed by immunoblotting for GAPDH.

Since we already knew that the ECM protein fibronectin is key in the regulation of cell fate and also has an important effect on invasive behavior of HPV8-E7 positive keratinocytes (Heuser et al., 2016), we first investigated how HPV8-E7 may alter the secretome of keratinocytes relevant for a crosstalk between epidermal keratinocytes and the ECM. Here, we cultivated both the N/TERT-8E7wt, N/TERT-8E7^L23A^ (which codes for the invasion deficient mutant) as well as the empty vector control on a fibronectin matrix and collected the cell culture supernatants after 96 h. In the mass-spectrometric analysis we unveiled 11 proteins that were exclusively differentially secreted in N/TERT-8E7 cells compared to control and N/TERT-8E7^L23A^ (Figures 2A,B and Supplementary Table 2). A further GO enrichment analysis pointed toward most of the identified proteins (except for GALNT2 and UGP2) to be involved in processes associated with cell growth and proliferation (Figure 2C). Interestingly, 14-3-3 family members, which are known to be scaffolds that facilitate a multitude of protein-protein interactions, were present in these pathways and may therefore represent an important regulatory axis. In order to check if the reduced quantity of 14-3-3 in the secretome was the result of decreased protein expression we next performed Western blots with cell extracts of the same cells. Here, we could again show that the presence of HPV8-E7 had a suppressive effect on 14-3-3 β expression (Figure 2D).

**FIGURE 3:**
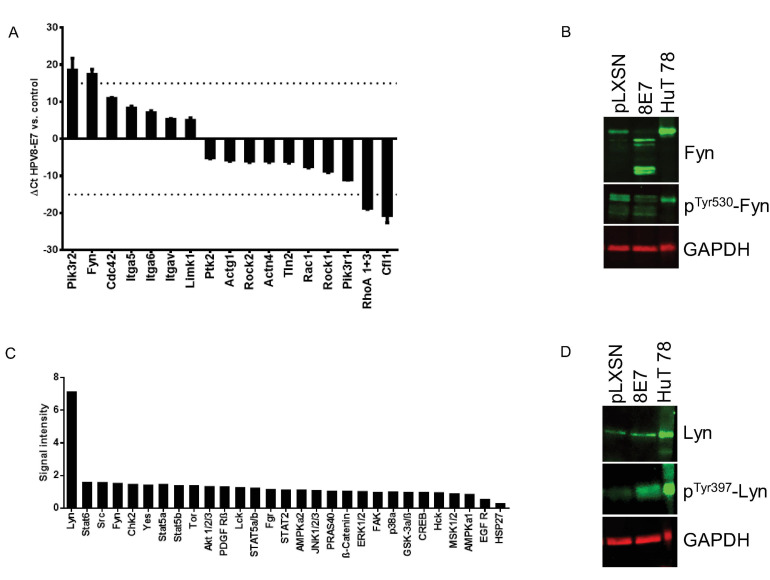
Analysis of fibronectin associated gene expression and protein kinase phosphorylation changes in HPV8-E7 positive keratinocytes. **(A)** PrimePCR results for N/TERT-8E7 grown on fibronectin for 96 h (*n* = 4). **(B)** Representative immunoblots of RIPA extracts from N/TERT-8E7 tested for total Fyn and p^Tyr530^-Fyn (*n* = 3). HuT78 whole cell lysates served as a positive control. Equal loading was confirmed by immunoblotting for GAPDH. **(C)** Protein kinase phosphorylation patterns in N/TERT-8E7 cells cultured on fibronectin for 96 h were analyzed using the Proteome Profiler Human Phospho- Kinase Array Kit (RnD Systems). The cut-off value for up- and down-regulated genes was set to fivefold. The dashed line indicates the 15-fold change. **(D)** Representative immunoblots of RIPA extracts of N/TERT-8E7 tested for total Lyn and p^Tyr397^-Lyn (*n* = 3). HuT78 whole cell lysates served as a positive control. Equal loading was confirmed by immunoblotting for GAPDH.

To narrow down on gene expression changes of known fibronectin-associated genes in HPV8-E7 positive keratinocytes we next performed PrimePCRs using the Fibronectin-Binding Integrin in Cell Motility H96 assay. These RTqPCRs were performed in quadruplicate, with RNA derived from both control as well as HPV8-E7 positive N/TERT grown on fibronectin coated cell culture dishes for 96 h. The PrimePCR revealed four genes that were more than 15-fold up- or down regulated, namely PIK3R2 (phosphoinositide-3-kinase regulatory subunit 2), Fyn as well as RhoA (ras homolog family member A) and Cfl1 (cofilin 1), respectively (Figure 3A). Since Src kinase family members, like Fyn, are known to be key modulators of cancer cell invasion and metastasis including the skin (Serrels et al., 2009), we next performed Western blots and could confirm higher protein levels and observed four major splice variants. Interestingly, Fyn phosphorylation at pTyr530, which is a negative regulatory tyrosine residue for this kinase (Qayyum et al., 2012) was weaker in E7 cells compared to the empty vector control (Figure 3B).

This observation sparked the idea to have a deeper look at differentially phosphorylated kinases in HPV8-E7 positive keratinocytes. We therefore used a phosphokinase array kit (Proteome Profiler^TM^ Array) with total cell extracts from N/TERT-8E7 grown on fibronectin-coated tissue culture plates for 96 h. Based on the obtained experimental data we discovered that now particularly the Src kinase family member Lyn seemed to be strongly hyperphosphorylated in HPV8-E7 positive cells (Figure 3C). Subsequent Western blot analyses proved that Lyn was indeed hyperphosphorylated at residue Tyr397 in HPV8-E7 positive keratinocytes whilst total protein levels remained unchanged (Figure 3D). Taken together, our findings show that Src-kinases are deregulated in HPV8-E7 positive keratinocytes grown on fibronectin.

### Identification of Differentially Phosphorylated Proteins in HPV8-E7 Positive Cells

**FIGURE 4:**
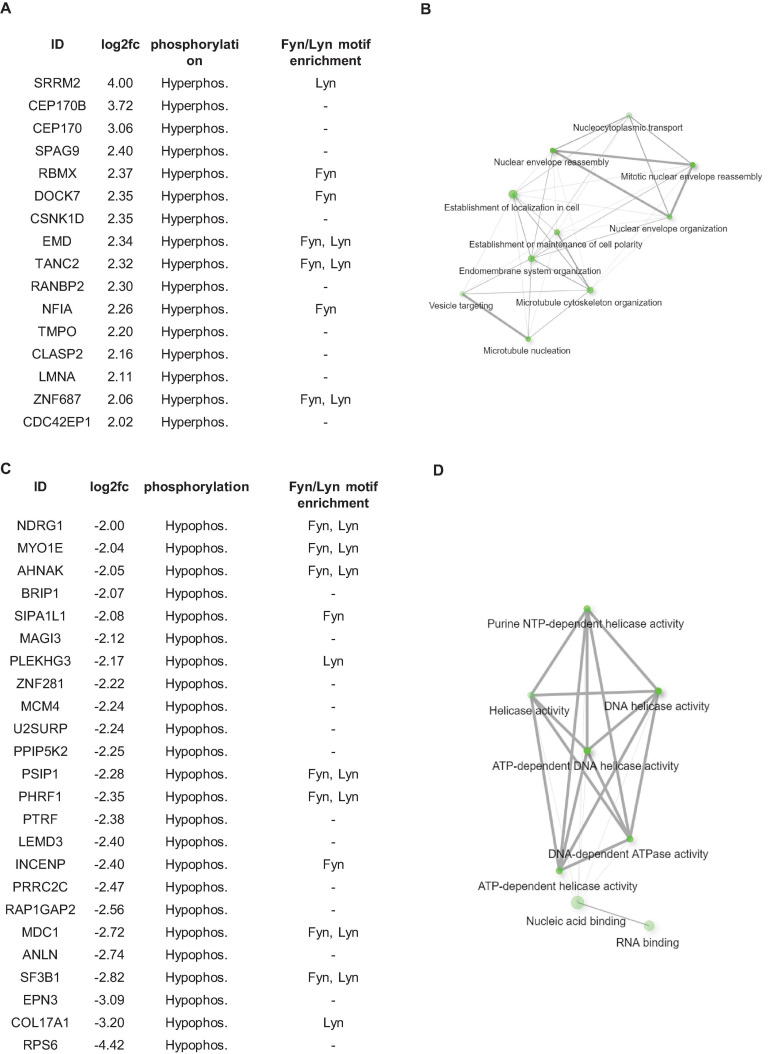
Phospho-proteome analysis of N/TERT-8E7 cultured on fibronectin. **(A)** List of proteins found to be hyper-phosphorylated in between HPV8-E7 positive keratinocytes. **(B)** Gene Ontology Enrichment Analysis of hyper-phosphorylated proteins. **(C)** List of proteins found to be hypo-phosphorylated in between HPV8-E7 positive keratinocytes. **(D)** Gene Ontology Enrichment Analysis of hypo-phosphorylated proteins. Fyn and Lyn kinase substrates identified using KinasePhos2.0 were highlighted in **(A,C)**.

Considering that protein kinases appear to play an important role in downstream signaling from fibronectin in HPV8-E7 positive cells we next expanded our studies to investigate the phospho-proteome of these cells. We first performed total proteome and phospho-proteome analyses of HPV8-E7 positive cells compared to control cells using the Perseus software suite. After subtracting the total proteins from the phopho-proteins we report 40 statistically significant phospho-proteins in HPV8-E7 expressing keratinocytes. Out of these 40 proteins, 16 were hyperphosphorylated and 24 proteins hypophosphorylated (Figure 4). The Perseus output table, showing the kinase target motif enrichments, excluding the results for Fyn and Lyn kinases, additionally predicts 14-3-3 binding motifs for several hypo- and hyperphosphorylated proteins as presented in Supplementary Table 3.

To pinpoint potential Fyn and Lyn targets, we performed an additional kinase motif enrichment analysis using the online tool KinasePhos2.0 (Wong et al., 2007). This analysis revealed that a great number of the identified differentially phosphorylated proteins are targets of the Src kinase family members Fyn and Lyn which may point toward HPV8-E7 hijacking these kinases to enact post-translational modifications in HPV8 infected cells (Figures 4A,C).

These aberrantly phosphorylated proteins could be grouped into two enriched pathway clusters, namely “cytoskeletal organization and cell polarity” and “DNA replication and repair” (Figures 4B,D). STRING database analyses further revealed that a majority of the identified proteins form protein-protein interaction networks (Supplementary Figure 1).

## Discussion

HPV8-E7 is the main viral oncoprotein exerting control over both proliferation and invasive behavior of keratinocytes (Akgül et al., 2005, 2007; Heuser et al., 2016; Hufbauer and Akgül, 2017). More detailed molecular insights on the mechanisms involved would therefore be invaluable to further expand our understanding of how these invasive processes are initiated and propagated. In order to shed more light on these issues this comprehensive study employed several different unbiased experimental angles, including transcriptomic, secretomic and proteomic/phospho-proteomic approaches.

From previous studies we already knew that a broad range of genes deregulated in HPV8-E7 positive primary keratinocytes are controlled upstream through Sp1/Sp3 binding sites in the promoter region (Kirschberg et al., 2019). Here, we now compared our data with a data set generated in the Alonso laboratory, with both data sets having been generated with either PHAK or PHFK, both grown on plastic. We thus identified three genes, namely ATF3, GADD34, and GDF15, as they showed the same expression pattern in the cDNA data sets of both studies. By means of RTqPCR we could validate both GADD34 and GDF15 as novel gene targets of E7 but not ATF3. One possible reason for this discrepancy could be that ATF3 might only be specifically targeted in primary keratinocytes but not in a cell line. Interestingly, the upregulation of GADD34 and GDF15 could only be detected in keratinocytes grown under undifferentiating conditions (grown in KGM-Gold), thus confirming these genes as novel gene targets of HPV8-E7. Despite the fact that both genes are known to be regulated by Sp1/Sp3 the exact mechanism by which this upregulation is achieved in HPV8-E7 positive cells should be further researched in future studies.

E7 co-expression with E6 even had a synergistic effect on GDF15 over-expression under undifferentiating conditions. In contrast, co-expression had a repressive effect on GADD34, whose expression was, however, still highly elevated compared to the control. When isogenic keratinocytes were grown under differentiating conditions the activating effect of E7 on GDF15 expression was abolished. Under these conditions, the GADD34 transcripts could not be detected at all pointing toward an exclusive role in undifferentiated keratinocytes. GDF15 is a member of the TGF-β superfamily. However, its role in keratinocyte biology has thus far not been extensively studied. It is known that GDF15 overexpression in melanoma cells is associated with tumor invasion and metastasis (Unal et al., 2015). In line with our data, it is reasonable to speculate that the E7 mediated upregulation and secretion of GDF15 may disturb the physiological keratinocyte differentiation program. This may also have an influence on epidermal-dermal crosstalk, which may ultimately result in aberrant cell growth. GADD34, on the other hand, belongs to a family of genes which are induced by DNA damage and apoptotic cell death, and which are also known to enhance the apoptotic response to DNA damage (Grishin et al., 2001). In later studies, GADD34 was shown to dephosphorylate several kinases that function in various important signaling cascades (Collier et al., 2017). Our observation that GADD34 is only detectable and thus seemingly targeted by E7 in undifferentiated keratinocytes with a basal cell phenotype points toward a role of this regulation axis in manipulating the growth of undifferentiated basal keratinocytes.

Since previous results of our group had shown the importance of fibronectin in modulating the phenotype of HPV8-E7 expressing keratinocytes we now aimed at identifying signaling molecules and cascades that are altered under these conditions.

Our secretomic and proteomic data both hint at a possible involvement of 14-3-3 family members in HPV8-E7 mediated cellular changes. The 14-3-3 family is comprised of a number of scaffolding proteins that serve as platforms for several protein-protein interactions. The 14-3-3 protein family fulfills various regulatory functions involved in signaling pathways, cell proliferation, DNA damage response, apoptosis, differentiation and cell survival. By altering key regulatory proteins 14-3-3 family members play pivotal roles in cancer initiation and progression (Khorrami et al., 2017; Liu et al., 2021). It is therefore not unreasonable to assume that the observed E7-mediated changes in 14-3-3 protein levels as well as changes in global protein phosphorylation patterns may also lead to changes in 14-3-3 binding motif availabilities as well. Considering that we found a downregulation of 14-3-3 protein levels both in cell culture supernatants as well as in full cell lysates our experimental results may thus have unveiled a modulatory axis hijacked by the HPV8-E7 oncoprotein which may contribute to disturbing physiological keratinocyte functions.

Using different experimental approaches and bioinformatical tools we identified the Src kinase family members Fyn and Lyn, among others (Figures 3, 4 and Supplementary Table 3), as novel crucial kinases deregulated by E7 that may play a vital role in altering the phosphorylation profile of cellular proteins involved in cytoskeletal organization and cell polarity as well as proteins associated with DNA replication and repair, which may ultimately contribute HPV8-E7 mediated keratinocyte transformation.

In that light, Src kinases are comprised of a family of 11 non-receptor tyrosine kinases and interestingly only Fyn and Lyn seem to become over-abundantly activated in HPV8-E7 positive keratinocytes grown on fibronectin. Here, Lyn expression has also been implicated to be activated in response to DNA damage. Also, Lyn—but not other Src kinases—is activated following genotoxic stress, where Lyn is associated with cell cycle arrest and apoptotic processes (Yoshida et al., 2000). Furthermore, Lyn is known to act as a negative regulator of GADD34 in DNA damage-induced cell death (Grishin et al., 2001). As in HPV8-E7 positive cells Lyn is overly activated one would expect now that GADD34 expression would also be repressed (as described above). Surprisingly, this does not seem to be the case, which points toward an uncoupling of this regulatory circuit in the presence of E7 and E6E7, respectively. Moving on to Fyn, this kinase has three known splice variants, namely FynT, FynB, and FynC, which arise from alternative splicing of exon 7 of the Fyn gene (Brignatz et al., 2009; Uddin et al., 2020). The observation of a fourth Fyn signal in Western blots of HPV8-E7 positive cells grown on fibronectin may potentially have unveiled a fourth unknown Fyn splice variant, which may be associated with E7 expression and contribute to the complexity of Src kinase regulation in betaHPV positive skin. Interestingly, in a previous study on HPV16-E7 the authors also described phosphorylation of Src family kinases Yes and Fyn in the presence of HPV16-E7 (Szalmas et al., 2013).

In addition to the already known HPV8-E7 binding partners (Enzenauer et al., 1998; Rozenblatt-Rosen et al., 2012; Sperling et al., 2012; White et al., 2012; White and Howley, 2013; Grace and Munger, 2017; Oswald et al., 2017, 2019) we now identified novel cellular targets which are differentially regulated in HPV8-E7 positive keratinocytes. The finding that the Src kinases Fyn and Lyn are significantly over-activated and likely change the phosphorylation status of several proteins when cells are grown on fibronectin may possibly represent the most profound results of our study. As Src kinase inhibitors already exist our results may therefore pave the way for novel therapeutic approaches in which these inhibitors could be used for treatment of skin SCCs in which betaHPV involvement has been demonstrated.

## Data Availability Statement

The original microarray data presented in the study have been deposited in the Gene Expression Omnibus (GEO) repository, accession number (GSE133813). The proteomics data for differentially phosphorylated proteins as well as for the secreted proteins are available at the ProteomeXchange repository, accession numbers (PXD026099) and (PXD026100), respectively.

## Author Contributions

MK, ASS, HGD, SH, AW-H, AA, and MH performed the experiments. MK, ASS, HGD, SH, AW-H, AA, MH, and BA analyzed the data. MK and BA designed the experiments and wrote the manuscript. All authors contributed to the article and approved the submitted version.

## Conflict of Interest

The authors declare that the research was conducted in the absence of any commercial or financial relationships that could be construed as a potential conflict of interest.

## Publisher’s Note

All claims expressed in this article are solely those of the authors and do not necessarily represent those of their affiliated organizations, or those of the publisher, the editors and the reviewers. Any product that may be evaluated in this article, or claim that may be made by its manufacturer, is not guaranteed or endorsed by the publisher.

## Supplementary Material

Supplementary Figure 1 STRING protein-protein interaction network. Interaction network of hyper-phosphorylated and hypo-phosphorylated protein targets of HPV8-E7. All nodes indicate the proteins and lines indicate the interaction between two proteins.

Supplementary Table 1 CDNA microarray data analysis from the Alonso group, DKFZ, Heidelberg.

Supplementary Table 2 Precompiled list of the secretome analysis.

Supplementary Table 3 Precompiled list of the phospho-proteome analysis.

## Abbreviations

ECM: extracellular matrix
PHAK: primary human adult keratinocyte
PHFK: primary human foreskin keratinocyte
DED: de-epidermalized human dermis.
