# Supplementary figures and images for: Novel Insights Into Cellular Changes in HPV8-E7 Positive Keratinocytes: A Transcriptomic and Proteomic Analysis
Source: Front Microbiol. 2021 Sep 6;12:672201. doi: 10.3389/fmicb.2021.672201 (PMC8450583; doi:10.3389/fmicb.2021.672201)

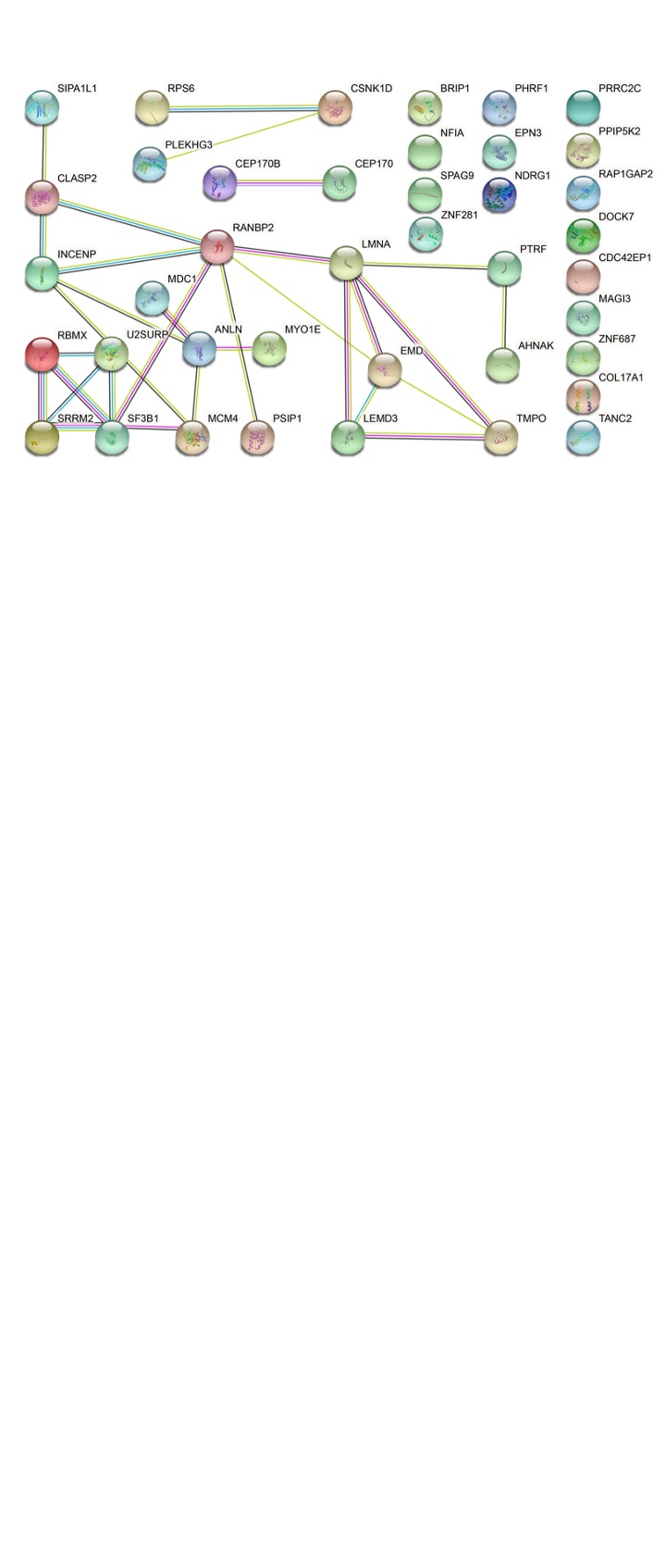

Supplement: Supplementary Figure 1 — STRING protein-protein interaction network. Interaction network of hyper-phosphorylated and hypo-phosphorylated protein targets of HPV8-E7. All nodes indicate the proteins and lines indicate the interaction between two proteins. [file Image_1.JPEG]
